# An Observational Study: Is *N*-Acetylcysteine Helpful in Performance Improvement of Mycoplasma IST2 Testing through Sample Homogenization?

**DOI:** 10.1155/2020/1391698

**Published:** 2020-07-02

**Authors:** Xin-Ru Mao, Rui-Cheng Wang, Rong-Jiao Li, Cai-Rong Zhou, Xian-Kai Chen, Can-Can Cheng, Xiao-Mao Yin

**Affiliations:** ^1^Clinical Laboratory, Nanfang Hospital of Southern Medical University, Guangzhou, China; ^2^Clinical Laboratory, Zhongshan Guzhen People's Hospital, Zhongshan, China; ^3^Clinical Laboratory, The Fifth Affiliated Hospital of Southern Medical University, Guangzhou, China; ^4^Clinical Laboratory, Guangzhou Panyu Central Hospital, Guangzhou, China; ^5^Department of Blood Transfusion, The Fifth Affiliated Hospital of Southern Medical University, Guangzhou, China

## Abstract

**Background:**

Culture is still the gold standard for the detection of genital mycoplasma which could cause urogenital infections in humans. Mycoplasma IST2 is a commercial kit widely used for the detection of *M. hominis* and *Ureaplasma* species. Its accuracy was partially impaired because clinical specimens are usually mixed with purulent or transparent mucus. We aimed to solve this problem through sample homogenization by *N*-acetylcysteine (NAC) treatment.

**Methods:**

Twenty-two endocervical swab samples were collected from 22 female patients with suspected mycoplasma infection, while 11 of these specimens were with purulent or transparent mucus. Mycoplasma IST2 testing kit was used for mycoplasma culture and AST for the control group and NAC-treated group.

**Results:**

Genital mycoplasma was detected in 15 of 22 samples for both groups. The colony number in 6 out of 11 purulent specimens (54.5%) was more than 10^4^ CFU/ml of genital mycoplasma for the NAC-treated group, while only one of 11 (9.1%) for the control group. For the nonpurulent specimens, no significant difference had been found in colony counting of genital mycoplasma between the control group and NAC-treated group (*P* > 0.05). The results of antimicrobial susceptibility testing for the NAC-treated group were highly similar to those for the control group.

**Conclusions:**

Our results demonstrate that NAC is helpful in sample homogenization and NAC treatment can improve the detection efficiency of mycoplasma with Mycoplasma IST2 testing.

## 1. Introduction

*Ureaplasma urealyticum*, *Ureaplasma parvum*, *Mycoplasma hominis,* and *Mycoplasma genitalium* are common urogenital pathogens, which could cause a large variety of infections in adults and infants [1]. The first two mycoplasma species are responsible for nongonococcal urethritis and bacterial vaginosis and are associated with postpartum fever, chorioamnionitis, low weight, and preterm birth [2]. The last two mycoplasma species are also causative agents of nongonococcal urethritis and have been associated with bacterial vaginosis, cervicitis, pelvic inflammatory disease (PID), postpartum septicemia, endometritis, and epididymitis [2].

Laboratory diagnosis of urogenital mycoplasma is usually by culture, but it may take 8 weeks for *M. genitalium* to grow into visible colonies [3]. Therefore, *M. genitalium* is detected mainly by nucleic acid amplification in clinical laboratory. There have been some commercial diagnostic kits for culture, identification, and antimicrobial susceptibility testing (AST) of urogenital mycoplasma species, such as Mycoplasma IES kit, Mycofast Revolution kit, Mycoplasma Duo kit, Mycoview kit, and Mycoplasma IST2 kit [1, 4]. These assays are based on liquid broth cultures and similar with regard to growth judgment by pH-sensitive color indicator. To our knowledge, all abovementioned assays could differentiate *M. hominis* from *Ureaplasma* species, but not differentiate *U. urealyticum* from *U. parvum*.

Mycoplasma IST2 is widely used for the detection of *M. hominis* and *Ureaplasma species*, including *U. urealyticum* and *U. parvum*. The broth of Mycoplasma IST2 contains arginine and urea which are supplemented with other essential nutrients that are required for the growth of mycoplasma. Urea is cleaved by urease for *Ureaplasma* species, and arginine is decomposed by arginase for *M. hominis*. Then, NH3 is released into the liquid medium, and the pH value of culture solution increases [5]. In addition, the culture medium is partially selective because it contains three antibacterial antibiotics and one antifungal drug, which could restrict the growth of most bacteria and fungi. The growth of mycoplasma with urea or arginine degradation in this medium is detected by the phenol red dye. The color of the indicator changes and indicates the growth of corresponding mycoplasma [6].

Urethral swab, urine, endocervical swab, and endometrial biopsy are appropriate specimens used for mycoplasma detection. Urethral swabs or endocervical swabs from female patients are usually mixed with purulent or transparent mucus, which are difficult to release pathogens wrapped in the mucous capsules. Because the mucous secretion cannot be completely dissolved and the clots are formed in the liquid medium, the release of mycoplasma is restricted and some incorrect results may appear in the subsequent colony counting and AST. To solve this problem, we tried to promote sample homogenization and mycoplasma release by adding digestion agent into the broth. *N*-Acetylcysteine (NAC) is the *N*-acetylated derivative of the natural amino acid *L*-cysteine, which could be used to loosen thick mucus as digestion agent with strong reducibility. It can digest mucus and homogenize the sample mainly by breaking the disulfide bonds and degrading the mucin [7]. As we know that NAC is also used as a common digestion-decontamination reagent for the processing of sputum for smears and cultures for mycobacteria. Our study aimed to evaluate the effect of NAC on Mycoplasma IST2 testing, and we found that NAC was helpful in mucus digestion and sample homogenization when used for Mycoplasma IST2 testing.

## 2. Materials and Methods

### 2.1. Clinical Samples

The study was conducted at the Fifth Affiliated Hospital of Southern Medical University from January 2017 to May 2020. Twenty-two endocervical swab samples were collected from 22 female patients with suspected mycoplasma infection. Eleven of these specimens were with purulent or transparent mucus, while the others were not. Patients that received antibiotics treatment within one week were excluded. All clinical samples were processed and cultured within 2 hours of collection.

### 2.2. Sample Processing

Mycoplasma IST2 testing kit was used for mycoplasma culture and AST (BioMérieux, Marcy l'Etoile, France). For the control group, swab specimen was inserted into R1 culture bottle which was filled with 3.1 ml of culture medium and vortexed the swab against the inner wall of the culture bottle for 30 seconds. It should be noticed that some purulent or transparent mucus were suspended in the solution, and some remained on the swab. Then, R1 solution was transferred into the R2 bottle and vortexed to dissolve the urea-arginine lyophilized pellet in the R2 bottle. After sample processing, all inoculated culture media were allowed to stand for 30 minutes at room temperature. For the NAC-treated group, the same swab specimen was inserted into another R1 culture bottle used for NAC digestion. The swab was vortexed, and the mucus was suspended in the medium. After mixing R1 broth with R2 reagent, 100 *μ*L of 0.5% NAC (adjusted pH to 6 with sodium bicarbonate) was added to the culture medium and all inoculated culture media were allowed to stand for 30 minutes at room temperature.

### 2.3. Mycoplasma IST2 Testing

After completing the above steps, 55 *μ*L of culture media was added into each of the 22 test wells on the R3 strip of Mycoplasma IST2 kit. Two drops of sterile mineral oil were added into each well. The strip was incubated at 36°C for 24–48 hours. Wells 1 to 3 were used for the identification of *Ureaplasma* species (not differentiating *U. urealyticum* from *U. parvum*) and *M. hominis*. Wells 4 to 5 were used for the counting of *Ureaplasma* species and *M. hominis*, respectively, which measured whether the concentration of the strain in the specimen was higher than 10^4^ CFU/ml. Wells 6 to 22 were used to perform the AST of nine antimicrobial agents including doxycycline (DOT), josamycin (JOS), ofloxacin (OFL), erythromycin (ERY), tetracycline (TET), ciprofloxacin (CIP), azithromycin (AZI), clarithromycin (CLA), and pristinamycin (PRI). All antimicrobial agents except the PRI were tested at two concentrations, namely, high-level concentration and low-level concentration. The result was regarded as resistant (R) when the growth was observed at both two concentrations of some antimicrobial agents. Conversely, the result was regarded as sensitive (S) when the growth was inhibited at both two concentrations of some antimicrobial agents. The result was regarded as intermediate (I) when the growth was inhibited at high-level concentration, while not at low concentration of some antimicrobial agents [1]. After incubation, color changes were observed for all wells, and color change from yellow to orange even red without turbidity indicated the growth of *M. hominis* and/or *Ureaplasma* species, whereas no color change means no growth of *M. hominis* and/or *Ureaplasma* species. The results of the counting of *Ureaplasma* species were read after 24 hours, and the remaining results were read 48 hours later.

### 2.4. Statistical Analysis

The statistical software SPSS 13.0 for windows (SPSS, Chicago, USA) was used for all statistical analyzes. McNemar's test was used to calculate significant difference between the control group and NAC-treated group. *P* values <0.05 were considered statistically significant throughout.

## 3. Results

### 3.1. Strain Identification of Mycoplasma Cultivation

A total of 22 samples were included in this study. There was no difference in the positive rate of mycoplasma cultivation between the control group and NAC-treated group. The results of strain identification were identical for both groups. Genital mycoplasma was detected in 15 of 22 samples (68.2%), including 11 samples positive for *U. urealyticum* (50%) and 4 samples positive for both *M. hominis* and *Ureaplasma species* (18.2%), while none positive only for *M. hominis*. Seven samples were negative for genital mycoplasma detection (31.8%).

### 3.2. Colony Counting of Mycoplasma Strain

The results of the influence of NAC on the colony counting of mycoplasma strain in purulent and nonpurulent specimens are shown in Tables 1 and 2, respectively.

For the purulent specimens, the colony number in 6 out of 11 specimens (54.5%) was more than 10^4^ CFU/ml of genital mycoplasma for the NAC-treated group, while only one of 11 (9.1%) for the control group. The details of colony counting of genital mycoplasma for the control group and NAC-treated group are displayed in Table 1. There was significant difference in colony counting of genital mycoplasma between the control group and NAC-treated group (*P* < 0.05).

For the nonpurulent specimens, no significant difference had been found in colony counting of genital mycoplasma between the control group and NAC-treated group (*P* > 0.05), and data are shown in Table 2.

### 3.3. Antimicrobial Susceptibility Testing

The results of AST for the control group of 11 purulent specimens are shown in Table 3. For *Ureaplasma*, a positive only specimen (*n* = 7), all strains (100%) were sensitive to JOS, ERY, CLA, and PRI; 6 strains (85.7%) were sensitive to DOT and AZI; 5 strains (71.4%) were sensitive to TET; and one strain (14.3%) was sensitive to OFL, while no strain was sensitive to CIP. For both *Ureaplasma* and *M. hominis* positive specimens (*n* = 3), all strains (100%) were sensitive to DOT, JOS, TET, and PRI and were resistant to OFL, ERY, CIP, and CLA. Besides, 2 strains (66.7%) were resistant, and one was (33.3%) intermediate to AZI.

The results of AST for the NAC-treated group of all samples were highly similar to those for the control group, and only the result of one *Ureaplasma* strain of the purulent specimens changed from sensitive to intermediate to JOS after NAC treating. No significant difference in AST of genital mycoplasma had been found between the control group and NAC-treated group (*P* > 0.05).

## 4. Discussion

Mycoplasmas belonging to the class Mollicutes are the smallest prokaryotes capable of self-replication [8]. Most mycoplasmas colonize many animals and plants, and only a few are pathogenic for humans [5]. Of these pathogens, *U. urealyticum*, *U. parvum*, *M. hominis*, and *M. genitalium* are commonly referred to as genital mycoplasmas because they are transmitted by sexual contact [5]. Traditional culture is considered the gold standard in the detection of *Ureaplasma* and *M. hominis*, while the laboratory diagnosis of *M. genitalium* is almost exclusively carried out using nucleic acid amplification tests as high failure rate and lengthy incubation times [9]. Mycoplasma IST2 is a commercially available diagnostic assay which is based on liquid culture and pH indicator. It is widely used for the detection and AST of *Ureaplasma* and *M. hominis* from urogenital samples. Urethral swabs and endocervical swabs are the most common specimens used for Mycoplasma IST2 testing, while they are usually mixed with purulent or transparent mucus which are difficult to be digested without special treatment. NAC is a kind of digestion reagent which is widely used for sputum treatment of mycobacterial culture. Thus, NAC was used to digest mucus from endocervical swabs in this study, in order to improve the performance of Mycoplasma IST2 testing for *Ureaplasma* and *M. hominis*.

A total of 22 samples were used for both normal processing (control group) and NAC digestion (NAC-treated group). Genital mycoplasma was detected in 15 of 22 samples for both groups, and there was no difference in the positive rate of mycoplasma cultivation between two groups. *Ureaplasma* was considered a part of normal genital flora with a colonization rate of 40–80% in previous studies [4, 10, 11]. The positive rate (68.2%) in this study was similar to previous studies. In addition, each *M. hominis* strain was isolated from clinical sample together with *Ureaplasma* species, the positive rate (18.2%) of *M. hominis* was lower than that of *Ureaplasma* in our study, and these results was similar to previous reports [12, 13]. For the purulent specimens, the colony number in 6 out of 11 specimens (54.5%) was more than 10^4^ CFU/ml of genital mycoplasma for the NAC-treated group, while only one of 11 (9.1%) for the control group, so there was significant difference in colony counting of genital mycoplasma between two groups. For the nonpurulent specimens, no significant difference had been found in colony counting of genital mycoplasma between the control group and NAC-treated group. This indicated that NAC treatment could increase colony number of genital mycoplasma owing to the release of genital mycoplasma enclosed in purulent or transparent mucus and had no impact on qualitative analysis of mycoplasma cultivation. Nevertheless, colony counting of genital mycoplasma is very important for clinical decision because 10^4^ CFU/ml of genital mycoplasma is the threshold concentration for considering the clinical significance of mycoplasma isolation [2, 14, 15]. Therefore, NAC treatment could elevate the positive rate of mycoplasma cultivation and improve the diagnostic accuracy of genital mycoplasma infection if 10^4^ CFU/ml was used as threshold concentration.

The results of AST for the NAC-treated group were highly similar to those for the control group, except that the result of one *Ureaplasma* strain of the purulent specimens changed from sensitive to intermediate to JOS after NAC treating. There was no significant difference in the results of AST between two groups. For *Ureaplasma,* a positive only specimen (*n* = 7) of the purulent samples, almost all isolates were sensitive to JOS, ERY, CLA, and PRI, most isolates were sensitive to TET, DOT, and AZI, and few isolates were sensitive to OFL and CIP. Similar AST results of clinical *Ureaplasma* isolates were reported in previous study from Hangzhou, China [16]. The high level of quinolone resistance was also reported in other countries such as Greece, Switzerland, and Italy [11, 12, 15, 17]. Tetracycline resistance was at low rate in China, Greece, Hungary, and Italy [12, 13, 15, 16], but at high rate in South Africa, the USA, and Cuba [17–20]. The low level of macrolide resistance was found in this study and an earlier study also from China, while the high level was seen in other countries and in a later study from China with a large number of semen specimens [17, 21]. The differences abovementioned might contribute to geographical distribution of drug-resistant strains and increased resistance rate of clinical strains in recent years. Of course, small sample sizes could be a major cause of AST differences between our study and other studies. For both *Ureaplasma* and *M. hominis* positive specimens (*n* = 3) of the purulent samples, it was difficult to analyze respective AST results of *Ureaplasma* and *M. hominis* exactly, while high level of macrolide resistance might contribute to natural resistance of *M. hominis*.

On the contrary, it is important to inoculate appropriate bacterial load for AST. The 10^4^–10^5^ CFU/ml inoculum is recommended for AST of genital mycoplasma [17, 22]. Although Mycoplasma IST2 kit has separate wells for differentiating inoculum concentration of ≥10^4^ CFU/ml, an incorrect result of AST such as false-sensitive result would be acquired for some specimens with purulent or transparent mucus due to sample nonhomogenization. It has been observed that mycoplasma strains do not grow at low concentration but grow at high concentration of some antimicrobial agents. Our study presented that NAC treatment could promote mucus degradation and sample homogenization, and this would guarantee that actual quantity of isolated mycoplasma was measured in colony counting well and equivalent quantity of mycoplasma isolate was added into each well of Mycoplasma IST2 strip. Thus, the accuracy of Mycoplasma IST2 testing could be improved by use of NAC treatment on clinical specimens. Nevertheless, commercial kits including Mycoplasma IST2 kit could not indicate when the inoculum concentration is >10^5^ CFU/ml, which would yield false-resistant results due to excessive bacterial load as high as 10^7^ CFU/ml [17]. Fortunately, the differences in colony counting of genital mycoplasma between the control group and NAC-treated group had few impacts on AST results between two groups in this study. This indicated that the inoculum concentration of each AST well for the NAC-treated group was >10^4^ CFU/ml but not as high as 10^7^ CFU/ml. Meanwhile, all inoculated culture media for both groups were allowed to stand for 30 minutes at room temperature, and this process would promote mucus digestion and pathogen release like NAC treatment, which was demonstrated by our subsequent experiments (data not shown). As mentioned above, the growth of mycoplasma could cause the pH of medium increase, which could be detected by the phenol red dye changing the medium color from yellow to orange or red when alkaline. Ensuring that the pH of the culture medium does not change after NAC treatment was a key point of this experiment. Therefore, we adjusted the pH value of NAC solution to 6 before being used and made sure the medium pH value of the NAC group is consistent with those of the control group before cultivation, which could avoid incorrect experimental results. Besides, Mycoplasma IST2 kit cannot separate results for *Ureaplasma* and *M. hominis* mixed cultures and does not use test concentrations and breakpoints recommended by Clinical and Laboratory Standards Institute [17, 22]. Because of abovementioned disadvantages of Mycoplasma IST2 testing, additional investigation was required to improve the accuracy of colony counting and AST further.

In conclusion, although Mycoplasma IST2 kit is widely used for genital mycoplasma detection in the world, the accuracy of this assay was partially impaired because clinical specimens especially endocervical swabs from female patients are usually mixed with purulent or transparent mucus. NAC is a digestion reagent which can digest mucus and homogenize the sample mainly by breaking the disulfide bonds and degrading the mucin. It was used to degrade mucus and homogenize inoculum in this study. Our results demonstrate that NAC is helpful in sample homogenization and NAC treatment can improve the detection efficiency of mycoplasma with Mycoplasma IST2 testing. Owing to some limitations of our study, further research is needed to increase the accuracy and improve the performance of available commercial kits for genital mycoplasma detection.

## Figures and Tables

**Table 1:** Comparison of colony counting between the control group and NAC-treated group of purulent specimens (*n* = 11).

NAC-treated group	Control group (*n* = 11)		
	Counting ≥ 10^4^ CFU/ml	Counting < 10^4^ CFU/ml or negative	Total
Counting ≥ 10^4^ CFU/ml	1	5	6 (54.5%)
Counting < 10^4^ CFU/ml or negative	0	5	5 (45.5%)
Total	1 (9.1%)	10 (90.9%)	11 (100%)

**Table 2:** Comparison of colony counting between the control group and NAC-treated group of nonpurulent specimens (*n* = 11).

NAC-treated group	Control group (*n* = 11)		
	Counting ≥ 10^4^ CFU/ml	Counting < 10^4^ CFU/ml or negative	Total
Counting ≥ 10^4^ CFU/ml	2	0	2 (18.2%)
Counting < 10^4^ CFU/ml or negative	0	9	9 (81.8%)
Total	2 (18.2%)	9 (81.8%)	11 (100%)

**Table 3:** Results of antimicrobial susceptibility testing for genital mycoplasma in the control group of purulent specimens.

Antibiotic	*Ureaplasma* (*n* = 7)						*Ureaplasma* + *M. hominis* (*n* = 3)					
	R		I		S		R		I		S	
	*n*	%	*n*	%	*n*	%	*n*	%	*n*	%	*n*	%
DOT	1	14.3	0	0	6	85.7	0	0	0	0	3	100
JOS	0	0	0	0	7	100	0	0	0	0	3	100
OFL	4	57.1	2	28.6	1	14.3	3	100	0	0	0	0
ERY	0	0	0	0	7	100	3	100	0	0	0	0
TET	2	28.6	0	0	5	71.4	0	0	0	0	3	100
CIP	6	85.7	1	14.3	0	0	3	100	0	0	0	0
AZI	0	0	1	14.3	6	85.7	2	66.7	1	33.3	0	0
CLA	0	0	0	0	7	100	3	100	0	0	0	0
PRI	0	0	0	0	7	100	0	0	0	0	3	100

## Data Availability

The data used to support the findings of this study are available from the corresponding author upon request.
